# The Failure of London County Council Administration

**Published:** 1904-07-23

**Authors:** 


### THE FAILURE OF LONDON COUNTY COUNCIL ADMINISTRATION.

THE evidence given, and Mr. Justice Darling's remarks, at the Guildford Assizes in the Horton Lunatic Asylum case, following as they do upon similar failures under London County Council government, should result in prompt and radical reform. If it were possible to prove, at a voluntary hospital or public charity, that the books had been cooked, that there was a wholesale waste of goods in nearly every department, that when the stock was taken over-stock was destroyed, that the whole establishment was overstocked, and that much food was given away or removed from the institution and sold by a private trader for his own benefit, that institution would be closed, because the public would refuse to support it. Yet all these things were stated in evidence as having occurred under the London County Council's administration at the Horton Asylum, and we are glad to see the jury found that this establishment was grossly mismanaged, and that the conduct of the persons responsible for its administration should be at once seriously inquired into. No wonder the judge passed a light sentence on the prisoners, holding their masters and superiors responsible, and approved the finding of the jury. Mr. Justice Darling made the statement that the institution was "criminal from top to bottom," and he would not be surprised to find that twenty-five persons connected with the asylum had been guilty of improper dealings with the property of the Council. It is essential in our view that some member of the London County Council should rise in his place and ask for a list of the committee and officers of the Horton Lunatic Asylum to be read out at the next meeting of the Council. The electors will then know the sort of service rendered by their representatives, who have permitted the criminal proceedings referred to by the judge; and no doubt in this way, and in this way only, will full justice be secured. The London County Council has posed as an honourable body entitled to the highest credit for its efficiency in every department. The Horton Asylum case proves that inefficiency is rampant in some of the great public institutions under its control. We refrain from further comment pending the next meeting of the London County Council, when the members of that body will have an opportunity of showing by the steps which are taken in regard to this matter how far and to what extent they are worthy of public confidence. Those members of the public who clamour for rate-supported hospitals should read the evidence of this case carefully, in order that they may realise the state of efficiency to which our hospitals might fall if they were all placed to-morrow under the municipal authority which has permitted the grave scandals referred to to continue and flourish.

